# Synchrony and exertion during dance independently raise pain threshold and encourage social bonding

**DOI:** 10.1098/rsbl.2015.0767

**Published:** 2015-10

**Authors:** Bronwyn Tarr, Jacques Launay, Emma Cohen, Robin Dunbar

**Affiliations:** 1 Department of Experimental Psychology, University of Oxford, South Parks Road, Oxford, OX1 3UD, UK; 2 School of Anthropology and Museum Ethnography, University of Oxford, 51/53 Banbury Road, Oxford, OX2 6PE, UK; 3 Wadham College, Parks Road, Oxford, OX1 3PN, UK

**Keywords:** dance, synchrony, social bonding, self–other merging, endorphins

## Abstract

Group dancing is a ubiquitous human activity that involves exertive synchronized movement to music. It is hypothesized to play a role in social bonding, potentially via the release of endorphins, which are analgesic and reward-inducing, and have been implicated in primate social bonding. We used a 2 × 2 experimental design to examine effects of exertion and synchrony on bonding. Both demonstrated significant independent positive effects on pain threshold (a proxy for endorphin activation) and in-group bonding. This suggests that dance which involves both exertive and synchronized movement may be an effective group bonding activity.

## 1. Introduction

All human cultures perform and enjoy forms of music and dance in a group setting [1]. Dancing involves people synchronizing their movements to a predictable, rhythmic beat (usually provided by music) and to each other. In this manner, dance is fundamentally cooperative in nature, and may have served the evolutionary function of encouraging social bonds, cooperation and prosocial behaviours between group members [2–5]. To date, empirical support for this social bonding hypothesis is based mainly on a link between synchrony (i.e. performing the same movement at the same time) and bonding [5].

Synchronization between people influences their subsequent positive social feelings towards one another: compared with asynchronous or solo conditions, participants who tap in synchrony report increased feelings of liking [6], interpersonal trust [7], willingness to help their tapping partner and heightened sense of being similar in personality [8]. Synchronized rocking in a chair [9], walking in step [10] and performing simple body movements in time with others and a metronome [5] also encourage prosocial tendencies. These effects are argued to be owing to a blurring of the perception of ‘self’ and ‘other’ leading to a bond between actors [11].

A possible mechanistic explanation for the social closeness that arises during these synchrony-based activities draws on the role of neurohormones, such as endogenous opioids [2]. Endorphins are associated with social bonding in a range of mammals [12]. In humans, shared neural networks are involved in processing physical and social pain (e.g. rejection versus inclusion: [13]), and the experience of endorphin-induced pleasure in a social setting may lead to positive associations with those present. It is likely that the endogenous opioid system (EOS) plays some role in the formation of human social bonds [14,15]. However, it has not yet been established whether social bonding following synchronization involves elevated endorphin levels.

In humans, opioids are released in response to low levels of muscular and physiological stress (e.g. during exercise: [16]). PET studies provide evidence that the euphoria and analgesia following exercise (the ‘runner's high’) are associated with EOS activity [17]. Because PET is difficult and stressful, pain threshold tests are a recognized proxy measure of endorphin levels [18]. Pain thresholds are elevated following synchronized exertive activities such as rowing [19–21] and active musical performance in a group [22], suggesting that exertive group synchronous activities can activate the EOS, although it should be noted that endocannabinoids likely also contribute to exercise ‘highs’ [23].

It is plausible that endorphin release during exertive synchronization may facilitate interpersonal social bonding that arises during dance [2]. Previous studies investigating synchrony and bonding have generally involved low exertive movements (e.g. finger tapping: [7–9]; rocking: [10]; simple arm movements: [6,11]), whereas studies investigating exertion and endorphin release [19–21] have not measured social bonding, and may have been confounded by inappropriate control conditions. As such, the effects of synchrony and exertion on endorphin release and associated social bonding have yet to be investigated. This study manipulated both synchrony and exertion separately to examine the independent and interacting effects on perceived social bonding and pain threshold.

## 2. Methods

Two hundred and sixty-four high school participants (164 girls; mean age 14.82 ± 2.289 s.d.) were recruited at local schools on Marajó Island, Brazil. Groups of three students (60 groups of mixed gender) were randomly allocated to one of four movement conditions (high exertion synchrony; high exertion partial synchrony; low exertion synchrony; low exertion partial synchrony). In synchrony conditions, all participants performed the same movements to the same music at the same time; partial synchrony involved participants performing different movements to the same music. Exertion was manipulated by having participants learn either full-body dance movements performed standing (high exertion condition) or small hand gestures performed seated (low exertion condition).

### (a) Dependent variables

Change in pain threshold is a commonly used proxy for EOS activation [14]. Pain was measured with steady inflation of a blood pressure cuff on the subject's non-dominant arm [19–21]; participants were asked to indicate when the pressure became uncomfortable (up to a maximum inflation of 300 mmHg), with the corresponding pressure value acting as the response variable.

Participants rated closeness towards the other participants in the testing group (‘in-group’) and their school class (‘out-group’) on seven-point Likert's scale, including an adapted version of the inclusion of other in self scale [24], questions about connectedness and trust [10], likeability [6] and similarity in personality [8]. A combined ‘prosociality index’ for the in- and out-group was created by averaging scores (Cronbach's *α* = 0.744). Participants also rated their mood and experience of the experiment (for details see electronic supplementary material, S1).

### (b) Procedure

Prior to the test session, a series of movements was taught to the participants as a group. During the test session, participants danced continuously for 10 min to instrumental music (average 130 bpm; electronic supplementary material, S2) played through Sony speakers. Participants stood (high exertion) or sat (low exertion) in a circle, facing inwards. A card displaying a list of the taught movements was placed in front of each participant (electronic supplementary material, S1), and they were instructed to perform the listed movements in order, changing when given a verbal cue, repeating the sequence as often as required.

In the synchrony condition, participants received identical cards and performed the same movements at the same time. In the partial synchrony condition, each participant had a different card, ensuring that no participants performed the same movement simultaneously.

### (c) Statistical analysis

Multilevel linear modelling was used to account for individual variation, repeated measures and structuring by group and class, and is appropriate when data are not normally distributed (as is the case for some variables: see electronic supplementary material, S1). The dependent variables measured before and after the movement session (pain threshold and prosociality index) were modelled using the fixed factors of time point (before versus after), synchrony condition (synchrony versus partial synchrony) and exertion condition (high versus low exertion), including interactions between these effects. *Post hoc* analyses indicated that there was a significant effect of gender (electronic supplementary material, S1) so gender was included as a covariate.

## 3. Results

For all conditions, there were no differences in participants' experience of the activity, their prior experience of music-based activities or how successful participants felt they had been on the task (electronic supplementary material, S1 and table S2). Additionally, there was no main effect of synchrony or exertion on change in positive or negative affect (electronic supplementary material, S1 and table S3).

**Figure 1.**
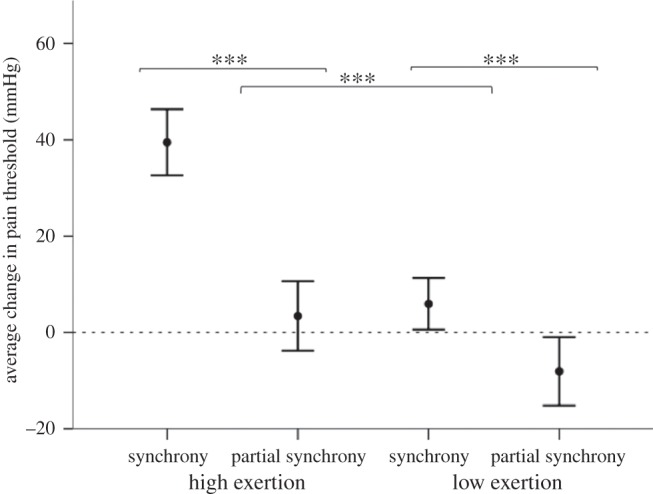
Mean (±1 s.e.) change in pain threshold in each movement condition. ***p ≤ 0.001.

There were significant positive main effects of both exertion (*F*_1_ = 11.310, *p* = 0.001) and synchrony (*F*_1_ = 13.978, *p* < 0.001) on change in pain threshold (i.e. end–start measure), with no interaction effect (*F*_1_ = 2.711, *p* = 0.101; figure 1).

**Figure 2.**
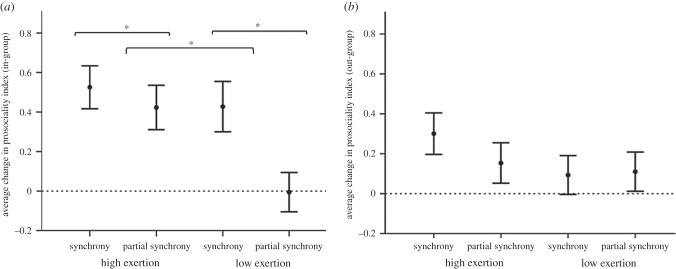
Mean (±1 s.e.) change for (*a*) in-group and (*b*) out-group prosociality index. *p ≤ 0.05.

Synchrony was associated with a significant increase in in-group prosociality ratings (*F*_1_ = 5.965, *p* = 0.015). Additionally, there was a significant main effect of exertion on in-group prosociality (*F*_1_ = 5.862, *p* = 0.016), with no interaction effect between synchrony and exertion (*F*_1_ = 2.325, *p* = 0.129; figure 2*a*). Synchrony and exertion did not affect out-group prosociality (figure 2*b*), although the out-group prosociality index was significantly higher after the activity overall (*F*_1_ = 11.503, *p* = 0.001; electronic supplementary material, S1 and table S4).

## 4. Discussion

This study aimed to elucidate the roles of synchrony and exertion on perceived social bonding and pain threshold following a naturalistic group dancing activity. Results demonstrate that both synchronization *and* exertion have positive independent effects on self-reported social bonding and pain threshold. Accordingly, findings previously restricted to non-exertive activities can generalize to everyday social activities, such as dance. We did not aim to investigate gender effects but found some differences between males and females (see electronic supplementary material, S1). Although this study includes only a subsample of one cultural group and we should perhaps be cautious in how we generalize these results across the species, we note that ethnographers have long observed, if only qualitatively, that dance has these bonding properties in a wide variety of cultures worldwide [1].

Although the link between movement synchrony and social bonding is well established, the role of the EOS and associated hormonal systems has not been previously investigated with regards to synchrony and exertion. In accordance with evidence from rowing studies [19–21], our results demonstrate a link between exertive, synchronous group movement and elevated pain threshold. Furthermore, we demonstrate that even low exertion tasks can result in elevated pain threshold when they are highly synchronized, and that synchrony and exertion have independent effects on this measure. Given that change in pain threshold is a widely used proxy for endorphin release, these findings suggest that the EOS is activated during synchronous activities, independent of the level of exertion, and may be an important link between synchrony and social bonding.

Previous evidence of social bonding between dyads performing simple movements in synchrony has focused on the mechanism of ‘self–other’ matching to explain social bonding [6,7,9]. When music and dance involve large groups of people, it is unlikely that they feel a sense of merging with all others present. Instead, it is more likely that the release of neurohormones causes some form of social ‘high’, which increases positivity towards those in the vicinity.

Notably, the social bonding effect was directed only towards fellow participants (the ‘in-group’), rather than towards absent but familiar members of the class (‘out-group’). Although previous studies have demonstrated that mimicked and synchronized movements can induce ‘generalized’ prosocial tendencies [25], we found no such effect. Even if this was owing to a small effect size, it remains the case that the prosocial effects experienced towards the out-group were not as substantial as those shown towards the in-group. Unlike previous work, our ‘out-group’ consisted of familiar others, who have presumably already been evaluated as potential friends, reducing the effect of any general increase in positivity. Social bonding and endorphin release during synchronized exertive movements are most pronounced with those who are present during the activity, and might also have more substantial effects with lesser-known others.

In so far as it might have direct or indirect fitness consequences, dance can be considered as an adaptive human behaviour [2], although we note that explicit links between dance and evolutionary fitness in humans have not yet been demonstrated. Here, we show that two key elements—synchronization and exertion—independently elevate pain thresholds and encourage bonding in a Brazilian sample. It is likely that additional features of dance (e.g. creativity, improvisation, ritual and cultural meaning) have also been honed over evolutionary history because they encourage a sense of cohesion, facilitating large-scale bonding and also because, as Darwin noted, they can have direct impacts on mate choice. More generally, activation of the EOS through synchronized behaviour might be instrumental in many social aspects of animal behaviour (e.g. the highly synchronized courtship rituals of grebes; [26]), and should be investigated further.

## Supplementary Material

ESM_1_Synchrony and Exertion

## Supplementary Material

ESM_3_Synchrony and Exertion_Data

## Data accessibility

All data are available in the electronic supplementary material.
