# Supplementary material for: Synchrony and exertion during dance independently raise pain threshold and encourage social bonding
Source: Biol Lett. 2015 Oct;11(10):20150767. doi: 10.1098/rsbl.2015.0767 (PMC4650190; doi:10.1098/rsbl.2015.0767)
Supplement: ESM_1_Synchrony and Exertion [file rsbl20150767supp1.docx]

## Electronic Supplementary Materials 1

## Questionnaires

##### Pre- and post- activity questions

In-group prosociality (where each question began with the words: “Thinking about all the pupils in this room now…”); and out-group prosociality (where each question began with the words: “Thinking about all the pupils in the whole class …”): questions: 1) Inclusion of Other in Self (IOS (1)): “…please choose the picture that best describes your relationship now.” (1 – 7 pictorial scale with labelled circles of increasing overlap to indicate relationship between ‘self’ and ‘group’); 2) Trust: “…How much do you trust the other pupils?” (2); 3) Connection: “…How connected do you feel to the other pupils?”(2); 4) Likeability: “…How likeable are the other pupils?” (2); and “…How much do you like the other pupils overall?” (3); 5) Similarity: “…Do you feel similar in personality to the other pupils?” (3). Questions 2 – 5 were assessed on a 7 – point Likert scale (where 1 = very slightly or not at all and 7 = extremely).

Pre- and post- questionnaires also included a short form of the Positive and Negative Affect Scale (PANAS): “Please indicate how you are feeling in this moment”, for distressed, excited, upset, scared, enthusiastic, alert, inspired, nervous, determined, and afraid (4).

##### Post- activity additional questions

Participants’ experience of the movement session was assessed by asking: “Now think about the movement session you just did and please answer the questions”: 1) Fun: “…Was the movement session fun?”; 2) Discomfort and embarrassment: “…How uncomfortable or embarrassed did you feel during the movement session?” ; 3) Difficulty: “…How difficult was the movement session for you?”; 4) Enjoyment: “…Did you enjoy the movement session?”; 5) Boredom: “…Were you bored during the movement session?” ; 6) Success: “…In your opinion, were you good at doing the movements?”,…”In your opinion were the other pupils good at doing the movements?”; “…In your opinion, were you in time with the music?”; and “In your opinion, were the other pupils in time with the music?” (Assessed on a 5 – point Likert scale where 1 = very slightly or not at all and 5 = extremely). The success questions were averaged to create a Success index (Cronbach’s α = 0.701).

Musicality/ dance experience was assessed by asking: 1) “Is music a part of your weekly life?”; 2) “Is dancing a part of your weekly life?” (Assessed on a 7 – point Likert scale where 1 = very slightly or not at all and 7 = extremely).

Hypothesis check: “What do you think this study is about? Write down your best guess”.

## Statistics

##### Normality testing and homogeneity of variance

Kolmogorov-Smirnov and Levenes’ tests indicated that the majority of the data were normally distributed with homogenous variance, although the trends were different for each of the dependent variables (see Table S1). Log transformations did not result in normality for any of the variables that were not normal.

Table S1. P-values for Kolmogorov-Smirnov normality test and Levene's test of homogenous variance on the start, end and change scores, and standardized residuals for each dependent variable.

| **Dependent variable** | **Movement condition** | **Start score (p-value)** | **End score (p-value)** | **Change (p-value)** | **Standardized residual of change (p-value)** | **Homogeneity of variance of change (p-value)** |
| --- | --- | --- | --- | --- | --- | --- |
| **Pain threshold** | High exertion synchrony | 0.034 | 0.000 | **0.093** | **0.066** | **0.172** |
|  | High exertion partial synchrony | 0.001 | 0.001 | 0.001 | 0.001 |  |
|  | Low exertion synchrony | 0.000 | 0.006 | 0.000 | 0.000 |  |
|  | Low exertion partial synchrony | 0.000 | 0.000 | 0.000 | 0.000 |  |
| **In-group prosociality index** | High exertion synchrony | 0.012 | **0.200** | **0.095** | **0.200** | **0.499** |
|  | High exertion partial synchrony | **0.200** | 0.013 | **0.200** | **0.200** |  |
|  | Low exertion synchrony | **0.200** | **0.200** | 0.000 | 0.000 |  |
|  | Low exertion partial synchrony | **0.073** | **0.082** | 0.020 | 0.040 |  |
| **Out-group prosociality index** | High exertion synchrony | 0.020 | **0.200** | 0.000 | 0.000 | **0.666** |
|  | High exertion partial synchrony | 0.030 | 0.034 | 0.004 | 0.007 |  |
|  | Low exertion synchrony | **0.052** | **0.200** | 0.004 | 0.010 |  |
|  | Low exertion partial synchrony | **0.200** | **0.200** | 0.004 | 0.003 |  |

##### Multilevel statistical modeling

Participant ID was added as a Level 1 random intercept (effectively accounting for the repeated measure), testing group was included as a Level 2 random intercept, and school class as a Level 3 random intercept. Gender was included as a covariate in all analyses, and as part of an unplanned post-hoc analysis, the male and female data were analysed separately (see below). Random effects were selected in the final models using backwards stepwise regression with log-likelihood tests to compare each model (via a χ^2^ analysis). Results are presented for the model of best fit, and in the event that the models were statistically equivocal, only the results for the model that included both individual and testing group as levels in the analysis are presented; although based on theoretical justification, the prosociality data were analysed with the further level of class included.

##### Additional statistical results

Table S2. Multilevel modeling results for participants’ experience of the movement session and their prior musical and dance experience.

|  |  | Sum of Squares | Df | Mean Square | F | *p* |
| --- | --- | --- | --- | --- | --- | --- |
| Synchrony | Fun | 1.662 | 1 | 1.662 | 2.699 | 0.104 |
|  | Embarrassed | 1.275 | 1 | 1.275 | 0.846 | 0.360 |
|  | Difficulty | 5.082 | 1 | 5.082 | 3.504 | 0.065 |
|  | Enjoyment | 0.603 | 1 | 0.603 | 1.375 | 0.244 |
|  | Bored | 4.025 | 1 | 4.025 | 2.484 | 0.119 |
|  | Success Index | 0.002 | 1 | 0.002 | 0.004 | 0.952 |
|  | Musicality | 0.115 | 1 | 0.115 | 0.025 | 0.876 |
|  | Ability to dance | 4.175 | 1 | 4.175 | 0.864 | 0.353 |
|  | Music activities | 2.803 | 1 | 2.803 | 0.887 | 0.349 |
|  | Dance activities | 0.049 | 1 | 0.049 | 0.011 | 0.918 |
| Exertion | Fun | 1.020 | 1 | 1.020 | 1.656 | 0.202 |
|  | Embarrassed | 0.081 | 1 | 0.081 | 0.054 | 0.817 |
|  | Difficulty | 1.012 | 1 | 1.012 | 0.698 | 0.406 |
|  | Enjoyment | 0.017 | 1 | 0.017 | 0.039 | 0.843 |
|  | Bored | 0.701 | 1 | 0.701 | 0.433 | 0.513 |
|  | Success Index | 0.496 | 1 | 0.496 | 1.117 | 0.294 |
|  | Musicality | 10.125 | 1 | 10.125 | 2.177 | 0.144 |
|  | Ability to dance | 3.548 | 1 | 3.548 | 0.735 | 0.392 |
|  | Music activities | 10.459 | 1 | 10.459 | 3.309 | 0.072 |
|  | Dance activities | 0.280 | 1 | 0.280 | 0.061 | 0.806 |
| Interaction | Fun | 0.012 | 1 | 0.012 | 0.020 | 0.888 |
|  | Embarrassed | 1.302 | 1 | 1.302 | 0.846 | 0.355 |
|  | Difficulty | 0.010 | 1 | 0.010 | 0.007 | 0.935 |
|  | Enjoyment | 0.025 | 1 | 0.025 | 0.057 | 0.811 |
|  | Bored | 0.018 | 1 | 0.018 | 0.011 | 0.916 |
|  | Success Index | 0.283 | 1 | 0.283 | 0.638 | 0.427 |
|  | Musicality | 12.406 | 1 | 12.406 | 2.668 | 0.106 |
|  | Ability to dance | 3.408 | 1 | 3.408 | 0.706 | 0.402 |
|  | Music activities | 0.001 | 1 | 0.001 | 0.000 | 0.984 |
|  | Dance activities | 14.501 | 1 | 14.501 | 3.144 | 0.080 |

Table S3. Results for repeated measures multilevel modeling of positive and negative affect.

|  | |  | Sum of Squares | Df | Mean Square | F | *p* |
| --- | --- | --- | --- | --- | --- | --- | --- |
| Time point | Positive affect | | 6.605 | 1 | 6.605 | 26.245 | <0.001 |
|  | Negative affect | | 7.920 | 1 | 7.920 | 36.283 | <0.001 |
| Time point *Synchrony | Positive affect | | 0.028 | 1 | 0.028 | 0.111 | 0.739 |
|  | Negative affect | | 0.001 | 1 | 0.001 | 0.003 | 0.955 |
| Time point *Exertion | Positive affect | | 0.002 | 1 | 0.002 | 0.007 | 0.932 |
|  | Negative affect | | 0.557 | 1 | 0.557 | 2.549 | 0.112 |
| 3-way Interaction | Positive affect | | 0.772 | 1 | 0.772 | 3.067 | 0.081 |
|  | Negative affect | | 0.146 | 1 | 0.146 | 0.671 | 0.414 |

Table S4. Results for repeated measures multilevel modeling of prosociality index (in- and out-group).

| PI |  | Sum of Squares | Df | Mean Square | F | *p* |
| --- | --- | --- | --- | --- | --- | --- |
| Time point | In-group | 14.476 | 1 | 14.476 | 35.453 | < 0.001 |
|  | Out-Group | 3.667 | 1 | 3.667 | 11.503 | 0.001 |
| Time point *Synchrony | In-group | 2.436 | 1 | 2.436 | 5.965 | 0.015 |
|  | Out-Group | 0.115 | 1 | 0.115 | 0.361 | 0.549 |
| Time point *Exertion | In-group | 2.394 | 1 | 2.394 | 5.862 | 0.016 |
|  | Out-Group | 0.445 | 1 | 0.445 | 1.340 | 0.239 |
| 3-way Interaction | In-group | 0.949 | 1 | 0.949 | 2.325 | 0.129 |
|  | Out-Group | 0.291 | 1 | 0.291 | 0.913 | 0.340 |

## Movement instruction cards

Each participant received a card detailing the order of movements (Figure S1).


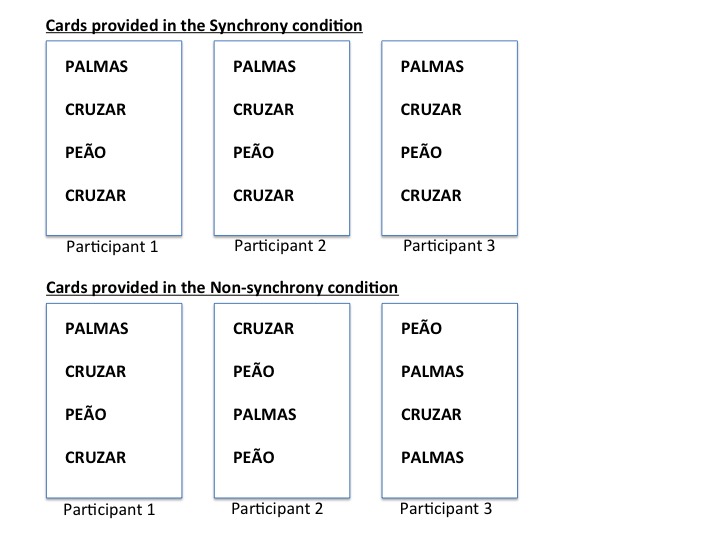


Figure S1. Cards detailing the order of movements for each participant in each movement condition.

##### High exertion movements

These movements were all performed standing. They included:

- -  ‘Palmas’ (translation: “clapping”): participants lifted their arms up and clapped their hands together (level with their face), then bent their knees and hit their hands on their thighs.
- -  ‘Peão’ (rough translation: “buffalo cowboy”): participants had their left arm placed across their chest and the right arm bent at the elbow, arm pointing upwards. They rotated their right upper arm (as if swinging a lasso), and moved their feet as if imitating a galloping horse. This movement was based on a similar *Carimbo^[[1]](#footnote-1)^* dance move.
- -  ‘Cruzar’ (translation: “cross”): participants bent and straightened their knees on alternate beats while simultaneously crossing bent arms in front of their chest, and then opening the arms out.
- Low exertion movements
- These movements were named similarly as the high exertion movements, but were adapted to be performed while seated (without any lower body movement) in order to reduce the physical exertion load.
- -  ‘Palmas’: participants clapped their hands together (in line with their lower torso), and then hit their thighs.
- -  ‘Peão’: participants placed their arms in an identical position to that of the high exertion ‘Peão’, but they were instructed to point upwards with their right-hand index finger and make small rotations at the wrist (rather than rotate the whole upper arm).
- -  ‘Cruzar’: participants straightened their hands and crossed them in front of their lower abdomen, and then face, alternating which hard was on top.

## Effects of gender

In order to test for possible differences between boys and girls, the dataset was split (for the distribution of males and females in the various movement conditions see Table S5). Furthermore,

Table S5. Distribution of males and females across the movement conditions.

| Movement condition | Males | Females | Total |
| --- | --- | --- | --- |
| High exertion synchrony | 23 | 49 | 72 |
| High exertion partial synchrony | 24 | 42 | 66 |
| Low exertion synchrony | 28 | 38 | 66 |
| Low exertion partial synchrony | 24 | 36 | 62 |
| Total | 99 | 165 | 264 |

##### Pain threshold

For males, there was a significant main effect of synchrony on the change in pain threshold (*F*(1) = 4.701, *p* = 0.033), but no significant effect of exertion (*F*(1) = 0.002, *p* = 0.961), nor an interaction effect (*F*(1) = 0.061, *p* = 0.805; see Figure S2a). In contrast, for females there was a significant main effect of both synchrony (*F*(1) = 7.960, *p* = 0.005) and exertion (*F*(1) = 18.704, *p* < 0.001), with an interaction effect (*F*(1) = 5.404, *p* = 0.021; see Figure S2b). In accordance with previous literature (e.g. 5), boys average pain threshold at the start (mean 225.39 ± 74.56 s.d) was higher than girls (mean 195.62 ± 69.93 s.d; Table S6; *F*(1) = 9.773, *p* = 0.002).

Table S6. Average ± s.d start, end and change scores in each of the dependent variables for males and females.

| **Dependent variable** |  | **Males** | **Females** |
| --- | --- | --- | --- |
| Pain threshold | Start score | 225.39 ± 74.56 | 195.62 ± 69.93 |
|  | End score | 234.65 ± 75.14 | 208.11 ± 70.91 |
|  | Change | 9.25 ± 56.35 | 12.48 ± 57.36 |
| In-group prosociality index | Start score | 4.35 ± 1.13 | 4.51 ± 1.21 |
|  | End score | 4.62 ± 1.34 | 4.89 ± 1.23 |
|  | Change | 0.30 ± 1.07 | 0.39 ± 0.82 |
| Out-group prosociality index | Start Score | 4.51 ± 1.21 | 4.33 ± 1.15 |
|  | End score | 4.45 ± 1.08 | 4.61 ± 1.21 |
|  | Change | 0.39 ± 0.78 | 0.24 ± 0.80 |

##### Prosociality (in-group)

For males there was a significant main effect of synchrony (*F*(1) = 9.015, *p* = 0.003) on the change in in-group prosociality index scores, but no significant effect of exertion (*F*(1) = 1.216, *p* = 0.273), and no interaction effect (*F*(1) = 0.156, *p* = 0.694; Figure S3a). For females, there was a significant main effect of exertion (*F*(1) = 5.535, *p* = 0.020), but no effect of synchrony on this measure (*F*(1) = 0.243, *p* = 0.623) nor an interaction (*F*(1) = 2.195, *p* = 0.140; Figure S3b).

##### Prosociality (out-group)

There were no significant main effects of synchrony or exertion on the out-group prosociality index scores for either males or females. Females showed an significant overall effect of timing on their rating of out-group prosociality index (*F*(1) = 14.486, *p* < 0.001), though this effect was not dependent on the movement condition.


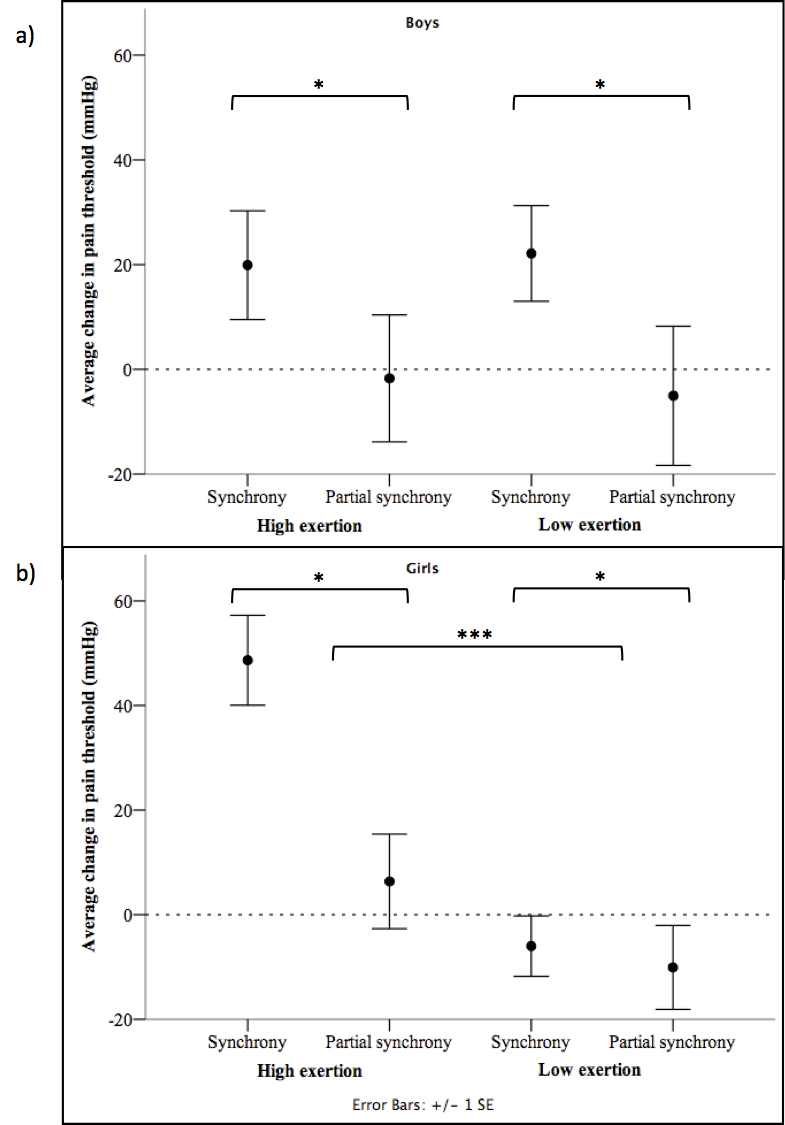


Figure S2. Mean (±1 SE) change in pain threshold in each movement condition for a) boys and b) girls. *** *p* ≤ 0.001, * *p* ≤ 0.05.


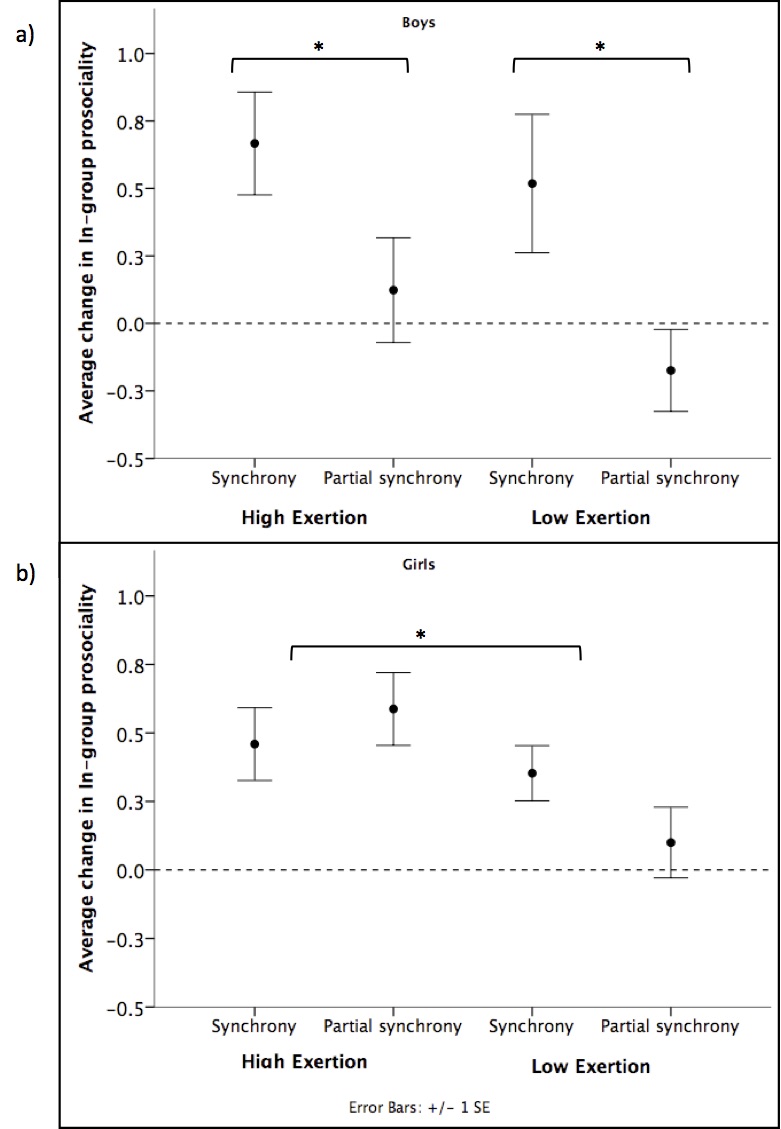


Figure S3. Mean (±1 SE) change in in-group prosociality index for a) boys b) girls. * *p* ≤ 0.05.

##### Discussion of gender effects

Although this study did not predict differences in the effects of synchrony and exertion on pain threshold and prosociality scores, these *post-hoc* analyses do indicate that there are differences between how boys and girls are affected in this group movement activity. For example, although the pain threshold increases significantly after synchronising compared to the partial synchrony condition for both boys and girls, exertion is an additional significant factor contributing towards this increase for girls, but not boys. These gender differences (and particularly the interaction effect between synchrony and exertion), is interesting but difficult to interpret, given that we did not, for example, collect data on levels of fitness (which may have differed between genders). For the prosociality data, girls’ prosociality scores were higher than that of boys (Table S6), and the differences between the genders on this measure may have been due to the fact that it was being measured on a 7-item scale, and so subject to potential ceiling effects.

The gender effects discussed above are interesting and certainly warrant investigation in future studies of the relative effects of synchrony and exertion. Nevertheless, these preliminary findings should be interpreted with caution, given that the sample sizes (especially for boys) were small (a consequence of the fact that gender was not an *a priori* consideration when designing this study). Interestingly, the synchrony literature reviewed in this paper do not highlight differences between men and women with regards to various prosociality measures. In terms of pain thresholds, there are well investigated differences between men and women (e.g. 5), but it is not clear how exertion and synchrony would be predicted to influence pain threshold per se. Future studies should investigate this by controlling for fitness, stage in menstrual cycle and whether groups have mixed genders or now.

References

*1. Aron A, Aron EN, Smollan D. Inclusion of Other in the Self Scale and the Structure of Interpersonal Closeness. J Pers Soc Psychol. 1992;63(4):596–612.*

*2. Wiltermuth SS, Heath C. Synchrony and Cooperation. Psychol Sci. 2009;20(1):1–5.*

*3. Valdesolo P, Desteno D. Synchrony and the social tuning of compassion. Emotion. 2011;11(2):262–6.*

*4. Mackinnon A, Jorm AF, Christensen H, Korten AE, Jacomb PA, Rodgers B. A short form of the Positive and Negative Affect Schedule: evaluation of factorial validity and invariance across demographic variables in a community sample. Pers Individ Dif. 1999;27:405–16.*

*5. Riley JL, Robinson ME, Wise E a., Myers CD, Fillingim RB. Sex differences in the perception of noxious experimental stimuli: A meta-analysis. Pain. 1998;74(2-3):181–7.*

1. *Carimbo* is a traditional dance of Marajó. It combines African beats with traditional Indian melody (performed by flute and song). ‘Peão’ refers to a man who rides horses to herd buffalo (which are farmed in the area as working animals and for meat/leather products). [↑](#footnote-ref-1)
